# Toad radiation reveals into-India dispersal as a source of endemism in the Western Ghats-Sri Lanka biodiversity hotspot

**DOI:** 10.1186/1471-2148-9-131

**Published:** 2009-06-11

**Authors:** Ines Van Bocxlaer, SD Biju, Simon P Loader, Franky Bossuyt

**Affiliations:** 1 Amphibian Evolution Lab, Biology Department, Unit of Ecology & Systematics, Vrije Universiteit Brussel (VUB), Pleinlaan 2, B-1050 Brussels, Belgium; 2 Systematics Lab, Centre for Environmental Management of Degraded Ecosystems (CEMDE), School of Environmental Studies, University of Delhi, Delhi, 110 007, India; 3 Institute of Biogeography, Department of Environmental Sciences, University of Basel, Klingelbergstrasse 27, 4056 Basel, Switzerland

## Abstract

**Background:**

High taxonomic level endemism in the Western Ghats-Sri Lanka biodiversity hotspot has been typically attributed to the subcontinent's geological history of long-term isolation. Subsequent out of – and into India dispersal of species after accretion to the Eurasian mainland is therefore often seen as a biogeographic factor that 'diluted' the composition of previously isolated Indian biota. However, few molecular studies have focussed on into-India dispersal as a possible source of endemism on the subcontinent. Using c. 6000 base pairs of mitochondrial and nuclear DNA, we investigated the evolutionary history and biogeography of true toads (Bufonidae), a group that colonized the Indian Subcontinent after the Indo-Asia collision.

**Results:**

Contrary to previous studies, Old World toads were recovered as a nested clade within New World Bufonidae, indicating a single colonization event. Species currently classified as *Ansonia *and *Pedostibes *were both recovered as being non-monophyletic, providing evidence for the independent origin of torrential and arboreal ecomorphs on the Indian subcontinent and in South-East Asia. Our analyses also revealed a previously unrecognized adaptive radiation of toads containing a variety of larval and adult ecomorphs. Molecular dating estimates and biogeographic analyses indicate that the early diversification of this clade happened in the Western Ghats and Sri Lanka during the Late Oligocene to Early Miocene.

**Conclusion:**

Paleoclimate reconstructions have shown that the Early Neogene of India was marked by major environmental changes, with the transition from a zonal- to the current monsoon-dominated climate. After arrival in the Western Ghats-Sri Lanka hotspot, toads diversified *in situ*, with only one lineage able to successfully disperse out of these mountains. Consequently, higher taxonomic level endemism on the Indian Subcontinent is not only the result of Cretaceous isolation, but also of invasion, isolation and radiation of new elements after accretion to the Eurasian mainland.

## Background

The Western Ghats of India and highlands of Sri Lanka are a global biodiversity hotspot, i.e. they contain several endemic animal and plant species which are recognised as being distinct at a high taxonomic level [1]. Endemism is particularly marked among amphibians, with over 75% of subcontinent species restricted to this region alone [2,3]. Explaining biogeographic patterns has mainly focussed on the geological history of the subcontinent, which indicates prolonged periods of isolation from the Late Cretaceous to the Early Tertiary [4-9]. Before joining the Eurasian mainland, India was part of Gondwana (South-America, Africa, Indo-Madagascar, Australia-New Guinea and Antarctica) and gradually became detached from other landmasses during its northward journey across the Tethys Sea [10-12]. Although the actual isolation of India has been debated (see [13] and references therein), molecular studies have demonstrated the presence of ancient lineages in India, and these are interpreted as evidence of this geological history [4-6].

The subsequent accretion of the Indian plate into Eurasia during the Early Tertiary [13] was a potential catalyst for biotic exchange between the subcontinent and the Eurasian mainland. It has been shown that an out-of-India dispersal of Gondwanan elements into Eurasia resulted in the wider distribution of previously isolated groups [5,14-17]. However, less attention has been given to the opportunity of "into-India" movement of Laurasian species after the establishment of this new geological connection. The exchange of species between the Indian Subcontinent and Eurasia must have been affected by the geomorphological changes generated after their collision. The uplift of the Himalayas and the Tibetan Plateau not only created physical barriers for dispersal, but also heavily influenced the prevalent climate [18,19]. The climatic regime in Asia changed from a zonal-pattern to a monsoon-dominant system, a reorganization that had major consequences for Asian and Indian terrestrial ecosystems [20,21].

True toads (Bufonidae) are a family of anurans with a wide distribution, comprising over 500 species [22,23]. Their present-day natural absence on several landmasses of Gondwanan origin (Australia, Madagascar, Seychelles), their nested position within Nobleobatrachia (Hyloidea *sensu *[6]), and their recent origin [24-26], imply that they reached the Indian subcontinent only after the Indo-Asia collision. Toads thus constitute an ideal group for studying Tertiary into-India dispersal. Despite numerous studies on phylogenetic relationships in Bufonidae [27-38], species from the Indian subcontinent were always underrepresented and their phylogenetic relationships remain largely elusive. In addition to multiple species whose taxonomic affiliations are uncertain (i.e., provisionally labelled "*Bufo*" pending more detailed phylogenetic evidence [37]), toads on the Indian subcontinent are classified in five genera: *Bufoides *(North-India) and *Adenomus *(Sri Lanka) are considered endemic to the subcontinent, *Duttaphrynus *has a wide distribution, and torrentially adapted *Ansonia *and arboreal *Pedostibes *species show disjunct distributions in wet-zone areas of South-East Asia and India [23]. Current taxonomy therefore suggests multiple dispersal events between the Indian subcontinent and adjacent regions.

Here we analysed a combined set of mitochondrial and nuclear gene fragments to investigate phylogenetic relationships and biogeography of Indian Bufonidae. Our sampling includes 15 toads from the subcontinent and a representative diversity of species of adjacent regions, with particular attention to shared genera *Ansonia*, *Pedostibes *and *Duttaphrynus*. We performed dating estimates and biogeographic analyses to investigate biotic exchange between the Indian subcontinent and adjacent regions, and evaluated how this affected endemism in the Western Ghats-Sri Lanka biodiversity hotspot.

## Results and discussion

### Phylogenetic relationships of Bufonidae

**Figure 1:**
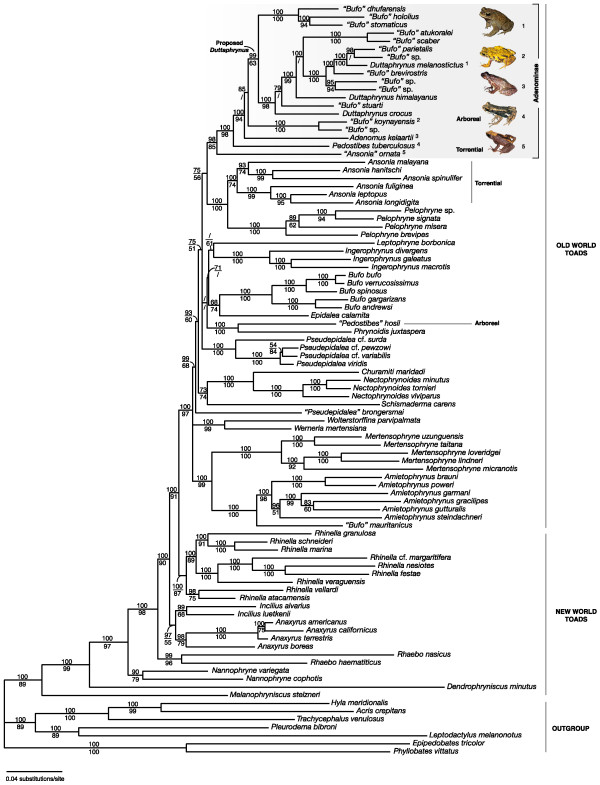
**Bayesian consensus phylogram for bufonid relationships**. Numbers above and below the branches indicate Bayesian Posterior Probabilities and Maximum Likelihood Bootstrap values, respectively. Pictures correspond to the ecomorphs included in the Adenominae radiation (grey box): picture 1, *Duttaphrynus melanostictus*, represents the genus *Duttaphrynus*; picture 2, *"Bufo" koynayensis*, a tropical, Western Ghats endemic species; picture 3, *Adenomus kelaartii*, a slender, stream inhabiting toad endemic to the highlands of Sri Lanka; picture 4, *Pedostibes tuberculosus*, a semi-arboreal toad endemic to the Western Ghats of India, and picture 5, *"Ansonia" ornata*, a fully torrential species endemic to the Western Ghats.

**Figure 2:**
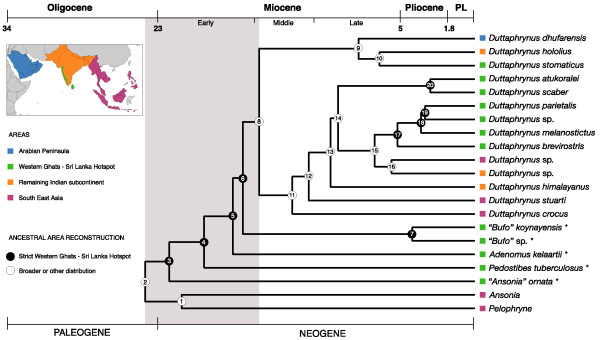
**Molecular timescale and ancestral area reconstruction for Adenominae**. Coloured rectangles indicate the area of sampling. Asterisks indicate lineages endemic to the Western Ghats-Sri Lanka (WG-SL) biodiversity hotspot. Circles on the nodes indicate ancestral area reconstructions (DIVA): black circles show ancestral species whose distribution is restricted to the WG-SL hotspot, white circles show ancestral species with a broader or other distribution. Exact reconstructions and divergence time estimates are given in Table 3, where node numbers are cross-referenced. The grey box indicates the timeframe between the nodes representing the earliest possible dispersal in, and latest possible dispersal out of the WG-SL biodiversity hotspot (DIVA analysis).

Our data matrix consists of 6309 basepairs (bp, 4339 bp mitochondrial- and 1970 bp nuclear DNA), 5116 bp of which could be unambiguously aligned. Maximum Parsimony (MP) analyses of the total dataset produced 2 equally parsimonious trees of 15224 steps. Maximum likelihood (ML) analyses of the total dataset produced a single ML tree (-lnL = 71238.66624, with gamma shape parameter = 0.533, and proportion of invariable sites = 0.483), which is very similar to the Bayesian consensus phylogram (Figure 1). Our analyses confidently determine the phylogenetic position of ten known species currently treated as *incertae sedis "Bufo" *in Figure 1). In agreement with previous evidence [31,32], we recover with high support the Northern African *"Bufo" mauritanicus *as sister species of a sub-Saharan *Amietophrynus *clade (Figure 1). Based on this support, we suggest transferring the species "*Bufo" mauritanicus *Schlegel, 1841 to the genus *Amietophrynus*. The remaining "*Bufo*" species in our study are nested in, or closely related to the genus *Duttaphrynus *as originally defined ([37], i.e. in essence the *Bufo melanostictus *group *sensu lato *[39,40], Table 1). The *Bufo scaber *group [39], here represented by *"Bufo" scaber *and *"Bufo" atukoralei*, is nested in *Duttaphrynus*. To restore monophyly of *Duttaphrynus *and to place sampled *"Bufo" *species in a taxonomic framework, we suggest expanding *Duttaphrynus *to include the most recent common ancestor of *Bufo melanostictus *(Schneider, 1799) and *Bufo stomaticus *Lütken, 1864, and all of its descendants. Eight known species, including those belonging to the *Bufo stomaticus *group *sensu *[39] (here represented by "*Bufo" dhufarensis*, *"Bufo" hololius *and *"Bufo" stomaticus*), are thus transferred to this genus (Figure 1, 2).

**Table 1:** Results of the AU test and Bayesian posterior probabilities for alternative hypotheses

Phylogenetic Hypothesis	- Ln L	Δ Ln L	P_AU_	BPP
Maximum Likelihood tree	71238.66624	Best		
Strict monophyly of *Ansonia*	71334.42926	95.76	<0.001	<0.001
Strict monophyly of *Pedostibes*	71308.29073	69.62	<0.001	<0.001
Strict monophyly of *Pseudepidalea*	71247.11034	8.44	0.302	<0.001
Strict monophyly of *Duttaphrynus *[37]	71480.10244	241.44	<0.001	<0.001

Δ ln L is the difference of likelihood between the ML tree and the best tree compatible with the constraint indicated under "Phylogenetic Hypothesis"

In contrast to previous studies, we recover with high support the Old World toads as a clade nested among New World taxa (Figure 1), but the sequence of early diversification of its major lineages remains poorly resolved. Furthermore, our analyses differ from previous hypotheses in a number of important aspects: First, the African species *Schismaderma carens*, in contrast to other studies [29-32,37,41], is recovered as sister species of a strongly supported *Churamiti*-*Nectophrynoides *clade. However, support for this African clade as a whole remains moderate. Second, in agreement with a previous study [42], our analyses do not support the monophyly of *Pseudepidalea *but find an ambiguous phylogenetic position for *Pseudepidalea brongersmai *(Figure 1; Table 1, P_AU _= 0.302, BPP < 0.001). Third, the genus *Pedostibes *is not recovered as being monophyletic (Table 1, P_AU _< 0.001, BPP < 0.001):*P. hosii *does not group with the type species *P. tuberculosus*, but comes out as the sister species of *Phrynoidis juxtaspera *(Figure 1). Fourth, species of the genus *Ansonia *similarly do not form a clade (Table 1, P_AU _< 0.001, BPP < 0.001). Instead, South-East Asian species group with *Pelophryne *(Figure 1).

### A previously unrecognized adaptive radiation of toads

Our analyses reveal a previously unrecognized adaptive radiation of toads, containing torrential, (semi-) arboreal, and drought-adapted ecomorphs in adult and/or larval forms (Figure 1, grey box). The name Adenominae Cope 1861 is available to denote this clade in a phylogenetic or Linnaean system [43]. The Malabar Torrent Toad "*Ansonia" ornata *from the Western Ghats is the sister lineage of all other genera in this radiation (Figure 1). Courtship, mating, egg-laying and tadpole development of this species all happens in and around torrential habitats. It also exhibits morphological characters that reflect its torrential ecology (i.e. toe webbing, slender body and long legs). Furthermore tadpoles of this species possess a specialized suctorial disk, allowing them to cling on rocks in fast flowing water (S.D. Biju, personal observation). Although both larvae and adults of the disjunctly distributed South-East Asian *Ansonia *species look remarkably similar [44], they form the sister clade of the Asian genus *Pelophryne*, species that show terrestrial adaptations (i.e., few large eggs and non-feeding tadpoles developing in small temporary pools or water-filled leaf axils [44-46]). The creation of a new genus will be necessary to accommodate the Indian species *Ansonia ornata *Günther, 1876 (unpublished data).

Another interesting member of this toad radiation is the Malabar Tree Toad *Pedostibes tuberculosus*, a Western Ghats endemic species that is largely arboreal [47]: During the night, males attract the females by calling from tree holes or an elevated position on leaves of vegetation. Furthermore, the fingers and toes of this species are dilated in broad truncated disks, which is a clear adaptation to their arboreal lifestyle. Our analyses indicate that *Pedostibes *from the Indian Subcontinent and the Malayan Peninsula independently evolved toe pads and an arboreal ecology [47,48]. *Pedostibes tuberculosus *also has a specialized development: it lays about 250 eggs in a clutch (*i.e*., not in strings, typical for many bufonid species) at the edge of streams, in which the bottom-feeding tadpoles further develop (S.D. Biju – personal observation).

Various ecomorphs are also evident in the genus *Adenomus*, which are slender toads endemic to the highlands of Sri Lanka (Figure 1) [49]. The three members, one of which (*Adenomus kandianus*) is considered extinct, inhabit streams in tropical montane forests or are semi-arboreal [49]. The radiation also includes a Western Ghats endemic lineage containing "*Bufo" koynayensis*, a species formerly placed in its own species group because its morphology did not correspond with any other recognized Oriental group [39]. This species often inhabits rocky mountain streams and has a bright yellow coloration during the mating season. Interestingly, it also lays its eggs in clutches (Varad Giri – personal communication). Because of these reasons, we suggest transferring "*Bufo" koynayensis *and its undescribed sister species to a new genus (unpublished data). Finally, the radiation includes a clade of pond-breeding species with a warty skin and more "typical" toad morphology. This group contains toads that were formerly placed in the *Bufo melanostictus, Bufo stomaticus*, *Bufo scaber *or *Bufo arabicus *groups. Together, they have a broad distribution, covering India, South-East Asia, the Arabian Peninsula, and possibly Northeast Africa.

### Dating estimates

Our dating estimates are listed in Table 2 and 3. Analyses using ingroup + outgroup calibrations (analysis A) and ingroup calibrations alone (analysis B) resulted in highly similar node ages. Additionally, removal of individual time constraints resulted in congruent dating estimates (data not shown). All analyses place the crown origin of Bufonidae around the Paleocene-Eocene border (analysis A: 55.35 ± 8.26 million years ago (Mya); analysis B: 54.49 ± 7.30 Mya, Table 2). This divergence time estimate is consistent with previous amphibian studies that cover a variety of methods and taxon sampling [24-26]. Our analyses further indicate that bufonids reached the Old World around 28.94 ± 4.43 Mya (Table 2). The origin of specialized endemic lineages (*"Ansonia"*, *Pedostibes*, the *"bufo" koynayensis *clade and *Adenomus*) on the Indian subcontinent is estimated between 22.42 ± 3.59 and 17.00 ± 2.86 Mya (Figure 2, nodes 3 to 6; Table 3).

**Table 2:** Divergence time estimates for relevant nodes in Bufonidae

	Dating estimates (Myr)			
	Mean ± SD	95% Interval	Mean ± SD	95% Interval
	Analysis A		Analysis B	

Stem Origin of Bufonidae	63.19 ± 9.32	[48.44, 84.57]	n.a.	n.a.
Crown Origin of Bufonidae	55.35 ± 8.26	[42.39, 74.26]	54.49 ± 7.30	[42.45, 70.97]
Stem Origin of Old World Clade	28.94 ± 4.43	[22.48, 39.50]	28.93 ± 3.94	[22.84, 38.02]
Crown Origin of Old World Clade	27.32 ± 4.21	[21.23, 37.44]	26.68 ± 3.62	[21.13, 34.98]
Stem origin of Adenominae	24.18 ± 3.79	[18.65, 33.46]	23.45 ± 3.23	[18.61, 31.02]
Crown origin of Adenominae	22.42 ± 3.59	[17.14, 31.05]	21.98 ± 3.08	[17.25, 29.12]

Mean ± standard deviation (Myr) and 95% credibility intervals (Myr) are shown for two sets of calibration points used in this study (analysis A and B, see main text)

**Table 3:** Dating estimates with corresponding ancestral distribution areas

			Analysis A		Analysis B	
Node N°	DIVA Reconstruction	MP Reconstruction	Mean ± SD	95% Interval	Mean ± SD	95% Interval
1	S	S	21.50 ± 3.43	[16.49, 29.68]	21.11 ± 2.98	[16.46, 28.06]
2	SW	S | W	24.18 ± 3.79	[18.65, 33.46]	23.54 ± 3.23	[18.61, 31.02]
3	W	W	22.42 ± 3.59	[17.14, 31.05]	21.98 ± 3.08	[17.25, 29.12]
4	W	W	19.86 ± 3.22	[15.08, 27.58]	19.20 ± 2.77	[14.89, 25.50]
5	W	W	17.74 ± 2.95	[13.34, 24.76]	17.19 ± 2.52	[13.27, 22.92]
6	W	W	17.00 ± 2.86	[12.69, 23.84]	16.75 ± 2.47	[12.92, 22.39]
7	W	W	4.56 ± 1.02	[2.94, 6.91]	4.65 ± 0.94	[3.12, 6.77]
8	W | SW | SWI | SWA | SWIA	W	15.81 ± 2.70	[11.74, 22.21]	15.66 ± 2.35	[11.97, 20.95]
9	WA | WIA	W	8.50 ± 1.67	[5.91, 12.35]	8.48 ± 1.48	[6.11, 11.84]
10	WI	W	6.97 ± 1.43	[4.71, 10.29]	7.05 ± 1.29	[4.94, 9.93]
11	S | SW | SWI	S | W	13.37 ± 2.36	[9.76, 18.95]	12.66 ± 2.01	[9.47, 17.35]
12	S | SW | SI | SWI	S | W	12.17 ± 2.19	[8.81, 17.33]	11.61 ± 1.87	[8.62, 15.85]
13	I | SI | WI | SWI	S | W | I	10.56 ± 1.96	[7.57, 15.15]	9.75 ± 1.65	[7.09, 13.47]
14	W | SW | WI | SWI	S | W | I	10.00 ± 1.88	[7.10, 14.43]	9.44 ± 1.61	[6.88, 13.09]
15	SW | WI | SWI	S | W | I	7.32 ± 1.44	[5.09, 10.62]	6.48 ± 1.18	[4.58, 9.19]
16	SI	S | W | I	6.08 ± 1.27	[4.08, 9.10]	5.33 ± 1.03	[3.66, 7.71]
17	W	W	5.64 ± 1.16	[3.82, 8.39]	4.96 ± 0.96	[3.42, 7.16]
18	W	W	3.92 ± 0.85	[2.58, 5.85]	3.41 ± 0.70	[2.29, 5.00]
19	W	W	3.62 ± 0.79	[2.36, 5.43]	3.17 ± 0.66	[2.11, 4.68]
20	W	W	3.24 ± 0.76	[2.03, 4.97]	2.70 ± 0.64	[1.66, 4.14]

Ancestral areas were reconstructed by both DIVA and MP analyses. Dating estimates of two sets of calibration points are shown (analysis A and B, see main text). Abbreviations are: S, South-East Asia; W, Western Ghats and Sri Lankan highlands; I, remaining Indian subcontinent; A, Arabian Peninsula. Nodes are cross-referenced in Figure 2

Our time estimates for the origin of bufonids differ substantially from a recent study where this event was placed in the Late Cretaceous (around 83 Mya) [30]. This older dating estimate can be attributed to the use of the oldest "leptodactylid" fossil *Baurubatrachus pricei*^† ^(86 Myr, [50]) to calibrate the crown node of Nobleobatrachia. This is possibly an overestimation: the identification of *Baurubatrachus *as a leptodactylid is questionable given the lack of synapomorphies defining this family [22] and its polyphyly according to molecular studies ([24] and references therein). For example, *Calyptocephalella gayi *was originally assigned to Leptodactylidae, but recent molecular studies have shown a close relationship to Australian Myobatrachidae. Therefore, calibrating the most recent common ancestor of all "Leptodactylidae", including *Calyptocephalella gayi *and Nobleobatrachia, at 86 Myr would have been a more conservative approach and would likely have given results similar to our dating estimates.

### Biogeography

Our trees show a deeply nested clade of Old World bufonids, indicating a single dispersal event (Figure 1). This result is in contrast with a previous hypothesis suggesting that one toad lineage returned to the New World [30]. Bayesian dating estimates suggest that bufonids reached the Old World around the Late Oligocene, a period that has been associated with global climate warming [51]. We hypothesize that this allowed toads to access northern latitudes and subsequently reach Eurasia. Although trans-oceanic dispersal cannot be excluded, colonization is more likely to have occurred over the Trans-Beringian land bridge, which connected Eurasia and North America intermittently from the Mid-Cretaceous until the late Pliocene [52,53]. After arrival in Eurasia, they rapidly spread southwards, reaching Africa, South-East Asia and the Indian subcontinent around the Oligocene-Miocene transition.

The early diversification of toads on the Indian subcontinent led to the origin of specialized endemic lineages (*"Ansonia"*, *Pedostibes*, the *"Bufo" koynayensis *group and *Adenomus*) in the Early Miocene (Figure 2). Dispersal-vicariance (DIVA) analyses (Table 3) indicate that this radiation occurred on the Southern parts of the subcontinent, as the oldest lineages are currently restricted to the Western Ghats and Sri Lankan highlands. The timing of this radiation corresponds with a period that is marked by global climatic- and environmental changes [18,54]. These were probably caused by the combined effect of Tibetan uplift and retreat of the Paratethys epicontinental sea, both of which were initiated by the Indo-Asia collision [20]. On the Indian subcontinent, the transition from a zonal to a monsoon-dominated climate pattern may have led to the aridification of the remaining peninsula, and a significant shift from closed forested tropical ecosystems towards more open savannah-like ecosystems [18,20,21,55-58]. If correct, specialized frogs distributed in the Western Ghats-Sri Lanka biodiversity hotspot would have become isolated from similar mountainous rainforest habitats. This would also explain why other rainforest clades radiated and largely remained in the highlands of Sri Lanka, even when sea-level variations frequently permitted dispersal to the mainland [59].

Our ancestral area reconstruction and dating estimates (Figure 2) indicate that around the Middle Miocene, toads successfully dispersed out of the Western Ghats-Sri Lanka hotspot and colonized other parts of the subcontinent, South-East Asia, and the Arabian Peninsula. It is possible that dispersal of these toads was more frequent, but more extensive taxon sampling is required to address this question.

## Conclusion

Our results show that the Late Oligocene-Middle Miocene interval was an important period for the diversification of specialized endemic lineages on the Indian subcontinent. It is likely that this period was not only essential for bufonid diversification, but also led to isolation of newly arrived lineages in other taxa of Laurasian origin. If confirmed, the Indian subcontinent's higher taxonomic endemism not only originated from Cretaceous isolation, but also from into India dispersal and subsequent radiation of new elements after accretion to the Eurasian mainland.

## Methods

### Taxon sampling and DNA protocols

This study includes 86 bufonid frog taxa representing the major lineages within the family, and a particularly comprehensive Indian sampling; 21 species served as outgroup taxa for dating estimates and 7 of these served as outgroup for phylogenetic analyes (see Additional file 1). DNA was extracted from muscle or liver tissue using a standard extraction protocol modified from Sambrook et al. [60]. Our total data matrix encompasses fragments of 2 nuclear genes (*NCX1 *and *CXCR4*) and 9 mitochondrial genes (*12SrRNA, tRNA^*VAL*^, 16SrRNA, tRNA^*LEU*^, ND1, tRNA^*ILE*^, tRNA^*GLN*^, tRNA^*MET*^, ND2*). Primers used in this study are published elsewhere [6,61-64]. PCR-products were purified following an agarose gel extraction protocol (Qiagen), cycle sequenced on both strands and analysed using a GeneScan 3100 automated sequencer. For 18 taxa, sequences were solely obtained from GenBank: ~2000 bp of mitochondrial (*12SrRNA *and *16SrRNA*) and ~700 bp of nuclear DNA (*CXCR4*) for 13 taxa, and ~2000 bp of mitochondrial DNA (*12SrRNA *and *16SrRNA*) for another 5 taxa (see Additional file 1, indicated with ^gb^).

### Sequence alignment and phylogenetic analyses

Sequences were aligned using ClustalX 1.64 [65]. Ambiguous sections were identified by eye for non-coding DNA and by comparison with amino acid sequences for coding DNA using MacClade v4.0 [66]. Sequences were deposited in GenBank, accession numbers are listed in Additional file 1. Phylogeny estimations were obtained under the maximum parsimony- and maximum likelihood criteria, and in a Bayesian framework. Heuristic MP searches were performed using PAUP* [67] and executed in 10000 replicates with all characters unordered and equally weighted, and using tree bisection reconnection branch swapping. ML searches were performed under the General Time Reversal (GTR) model, with substitution rates, gamma-shape parameter and proportion of invariable sites estimated from neighbour joining trees. These parameters were re-estimated from the best ML tree found so far, and this procedure was repeated several times. ML clade stability was estimated by non-parametric bootstrapping in 1000 replicates with PHYML 2.1b1 [68] using a GTR+G+I model of sequence evolution. Bayesian analyses were performed with MrBayes 3.1.2 [69], using a mixed model with four partitions: protein-coding mtDNA, RNA-coding mtDNA and the two nuclear gene fragments. Two runs of four Markov chain Monte Carlo (MCMC) chains each were executed in parallel for 10 million generations, with a sampling interval of 500 generations and a burn-in corresponding to the first five million generations. Convergence of the parallel runs was confirmed by split frequency standard deviations (<0.01), and by potential scale reduction factors (~1.0) for all model parameters using the software Tracer v1.3 [70]. Posterior probabilities for clades were obtained by combining the post-burn-in trees from parallel runs in a single consensus tree.

### Evaluation of alternative phylogenetic hypotheses

Alternative phylogenetic hypotheses represented by candidate trees estimated under ML using constraints in PAUP*, were compared using the approximately unbiased (AU) test [71]. Site-wise log-likelihoods for all trees were estimated using PAUP* and used as input for CONSEL 0.1 g [72]. Bayesian posterior probabilities for alternative phylogenetic hypotheses were estimated by screening the post-burn-in trees sampled by MrBayes using topological constraint filters in PAUP*.

### Divergence time estimates

We estimated nodal ages and 95% credibility intervals from our total DNA sequence data using MultiDivtime [73]. One single MCMC chain was run for 1.1 million generations, with a sampling frequency of one per 100 generations and a burn-in corresponding to the first 100,000 generations, assuming a F84+G model and an auto-correlated rate change. We calibrated our Bayesian consensus phylogram using five ingroup and two outgroup calibration points. Constrained nodes in the ingroup are: (A) A minimum age of 20 million years (Myr) for the split between North- and Central America based on the fossil *Bufo praevis *[74]. (B) A minimum age of 18 Mya for the stem origin of toads belonging to the *Bufo viridis *group based on several fossils belonging to this group: the Lower Miocene of Southeastern France, Greece [75], Northern Turkey [76] and Southern Germany [77]. (C) A minimum age of 11 Myr for the origin of *Rhinella marina *based on a fossil of this species from the Middle Miocene [78]. (D) A minimum age of 9.6 Myr for the origin of toads belonging to the *Bufo bufo *group based on the appearance of a *Bufo bufo *fossil from the Miocene of Europe (Czech Republic) [75]. The combined information of morphological species groups and high geographic structure in the phylogeny allowed using these fossils as conservative minimum age constraints.

Constrained nodes in the outgroup are: (E) A minimum age of 15 Myr for the stem origin of the subgenus *Eleutherodactylus *based on a fossil from northern Hispaniola [79,80]. (F) A minimum age of 35 Myr for the split between Phyllomedusidae – Pelodryadidae [81] corresponding to the last contact between the Australo-Papuan realm and the Neotropics [82].

We performed two analyses using different sets of calibration points as follows: (1) using all calibration points, referred to as analysis A and (2) using calibration points A-D, referred to as analysis B. For analyses A, the priors for the mean and standard deviation of the ingroup root age were set to 64.85 Myr and 12.25 Myr, respectively; for analyses B, the priors for the mean and standard deviation of the ingroup root age were set to 47.3 Myr and 9.8 Myr, respectively [24]. These ages define a fairly broad prior distribution for the origin op Nobleobatrachia and Bufonidae and cover the results of previous large-scale phylogenetic studies [24-26]. All estimates were done using the bayesian consensus phylogram; for analyses A, outgroup relationships were resolved according to previous phylogenetic evidence [24].

### Ancestral area reconstruction

Ancestral distributions were estimated on the Bayesian consensus phylogram under Maximum Parsimony using MacClade v4.0 [66] and under a dispersal-vicariance event based method implemented in DIVA 1.1 [83]. Both methods do not require a hypothesis of area relationships, but DIVA implements an explicit cost for extinction and dispersal events [83], for which default settings were used. We used four areas, corresponding to relevant biogeographic units: (1) South-East Asia (2) Western Ghats and Sri Lankan highlands, (3) remaining Indian subcontinent, (4) Arabian Peninsula. Taxa were coded based on their sampling locality, i.e. not on their presumed distribution. Although some species in *Duttaphrynus *(for example *Duttaphrynus melanostictus *here sampled in the Western Ghats) are not restricted to the coded area, this does not affect the general outcome of Early Miocene diversification of endemic genera in the Western Ghats – Sri Lanka biodiversity hotspot.

## Authors' contributions

IVB, SDB and FB conceived the study and designed the experiments. IVB and SPL gathered the molecular data. IVB performed the analyses. All authors collected samples and wrote the manuscript. All authors read and approved the final manuscript.

## Supplementary Material

Additional file 1 **Table S1. List of taxa included in this study with their sampling locality, corresponding tissue reference or voucher, and GenBank accession numbers**. * indicate species formerly treated as *incertae sedis *[23], ° indicate species for which a new genus will be described (unpublished data), names between quotation marks indicate provisional names, ‡ indicate species obtained from pet trade, gb indicates species for which ingroup sequences where solely obtained from GenBank. Ingroup taxa are ordered according to Figure 1. Collection abbreviations: BM, the Natural History Museum, London, United Kingdom; CAS, California Academy of Sciences, U.S.A; CEBB, Centre for Evolutionary Biology and Biodiversity, University of Adelaide, Australia; CMNHH Cincinnati Museum of Natural History, U.S.A; IZUA, Instituto de Zoología Universidad Austral de Chile, Chile; KU, University of Kansas, Museum of Natural History; KUHE, U.S.A; Kyoto University, Graduate School of Human and Environmental Studies, Japan; MNCN/ADN, Museo Nacional de Ciencias Naturales, Madrid, Spain; MTSN, Museo Tridentino di Scienze Naturali, Italy; MVZ, Museum of Vertebrate Zoology, Berkeley, U.S.A; QCAZ, Museo de Zoología, Pontificia Universidad Católica del Ecuador, Quito, Ecuador; ROM, Royal Ontario Museum, Toronto, Canada; TNHC, Texas Natural History Collections, Austin, U.S.A; USNM, National Museum of Natural History, Washington, U.S.A; UTA, University of Texas at Arlington, Department of Biological Sciences, U.S.A; VUB, Vrije Universiteit Brussel, Belgium; ZMMSU, Zoological Museum of Moscow State University Moscow, Russia. Collector abbreviations: DPL, Dwight P. Lawson; KMH, Kim M. Howell; MW, Mark Wilkinson; NP, Nickolai Poyarkov; SDB, S.D. Biju. Click here for file
