# Supplementary material for: Toad radiation reveals into-India dispersal as a source of endemism in the Western Ghats-Sri Lanka biodiversity hotspot
Source: BMC Evol Biol. 2009 Jun 11;9:131. doi: 10.1186/1471-2148-9-131 (PMC2706228; doi:10.1186/1471-2148-9-131)
Supplement: Additional file 1 — Table S1. List of taxa included in this study with their sampling locality, corresponding tissue reference or voucher, and GenBank accession numbers. * indicate species formerly treated as incertae sedis [23], ° indicate species for which a new genus will be described (unpublished data), names between quotation marks indicate provisional names, ‡ indicate species obtained from pet trade, gb indicates species for which ingroup sequences where solely obtained from GenBank. Ingroup taxa are ordered according to Figure 1. Collection abbreviations: BM, the Natural History Museum, London, United Kingdom; CAS, California Academy of Sciences, U.S.A; CEBB, Centre for Evolutionary Biology and Biodiversity, University of Adelaide, Australia; CMNHH Cincinnati Museum of Natural History, U.S.A; IZUA, Instituto de Zoología Universidad Austral de Chile, Chile; KU, University of Kansas, Museum of Natural History; KUHE, U.S.A; Kyoto University, Graduate School of Human and Environmental Studies, Japan; MNCN/ADN, Museo Nacional de Ciencias Naturales, Madrid, Spain; MTSN, Museo Tridentino di Scienze Naturali, Italy; MVZ, Museum of Vertebrate Zoology, Berkeley, U.S.A; QCAZ, Museo de Zoología, Pontificia Universidad Católica del Ecuador, Quito, Ecuador; ROM, Royal Ontario Museum, Toronto, Canada; TNHC, Texas Natural History Collections, Austin, U.S.A; USNM, National Museum of Natural History, Washington, U.S.A; UTA, University of Texas at Arlington, Department of Biological Sciences, U.S.A; VUB, Vrije Universiteit Brussel, Belgium; ZMMSU, Zoological Museum of Moscow State University Moscow, Russia. Collector abbreviations: DPL, Dwight P. Lawson; KMH, Kim M. Howell; MW, Mark Wilkinson; NP, Nickolai Poyarkov; SDB, S.D. Biju. [file 1471-2148-9-131-S1.doc]

| Genus | Species | Voucher / Tissue Ref | Locality | Genbank | | |
| --- | --- | --- | --- | --- | --- | --- |
|  |  |  |  | CXCR4 | NCX1 | Mitochondrial DNA |
| OUTGROUP |  |  |  |  |  |  |
| *Calyptocephalella* | *gayi* | MNCN/ADN 8002 | Chile | EF107495 | EF107275 | FJ882733-34 |
| Rhinoderma | *darwinii* | MVZ 164829 | Argentina | AY364192 | AY523733 | FJ882754-55 |
| *Batrachyla* | *taeniata* | MVZ 164828 | Argentina | AY948793 | AY948837 | FJ882752-53 |
| *Telmatobius* | *niger* | TNHC 62930 | South America | EF107464 | EF107239 | DQ347049, FJ882751 |
| *Ceratophrys* | *ornata* | VUB 1006 | South America | AY364188 | AY523718 | DQ347035, FJ882777 |
| *Trachycephalus* | *venulosus* | VUB 987 | South America | AY364185 | AY948824 | DQ347027, FJ882779 |
| *Acris* | *crepitans* | VUB 1085 | U.S.A | EF107468 | EF107244 | FJ882758-59 |
| *Hyla* | *arenicolor* | VUB 1052 | U.S.A | AY364190 | EF107241 | DQ347054, FJ882776 |
| *Hyla* | *meridionalis* | VUB 0534 | Spain | AY523687 | AY523710 | FJ882756-57 |
| *Pristimantis* | *cruentus* | MVZ 203826 | Costa Rica | AY948792 | AY948836 | FJ882746-48 |
| *Eleutherodactylus* | *coqui* | VUB 1624 | Puerto Rico | EF107500 | EF107282 | FJ882749-50 |
| *Eleutherodactylus* | *marnockii* | VUB 1036 | U.S.A | EF107463 | EF107238 | FJ882745 |
| *Phyllomedusa* | *hypochondrialis* | VUB 0990 | South America ^‡^ | AY948786 | AY948826 | FJ882741-42 |
| *Litoria* | *pulchra* | CEBB 45168 | Australia | AY948798 | AY948843 | FJ882739-40 |
| *Litoria* | *maini* | CEBB 35088 | Australia | EF107475 | EF107252 | FJ882737-38 |
| *Centrolene* | *prosoblepon* | MVZ 232752 | Panama | AY364193 | AY948834 | FJ882735-36 |
| *Leptodactylus* | *melanonotus* | MVZ 207294 | Costa Rica | AY364194 | AY948838 | FJ882762-63 |
| *Pleurodema* | *bibroni* | VUB 1030 | South America | AY948789 | AY948831 | FJ882760-61 |
| *Odontophrynus* | *occidentalis* | MVZ 145210 | Argentina | AY948791 | AY948835 | FJ882743-44 |
| *Epipedobates* | *tricolor* | VUB 1025 | South America ^‡^ | EF107458 | EF107233 | FJ882778 |
| *Phyllobates* | *vittatus* | VUB 1026 | South America ^‡^ | EF107459 | EF107234 | FJ882833-34 |
| INGROUP |  |  |  |  |  |  |
| *Duttaphrynus ** | *dhufarensis* | CAS 227584 | Oman | J882679 | FJ882626 | FJ882837-38 |
| *Duttaphrynus ** | *hololius* | SDB 4240 | India | J882680 | FJ882627 | FJ882781 |
| *Duttaphrynus ** | *stomaticus* | SDB 4020 | India: Western Ghats | J882681 | FJ882628 | FJ882787 |
| *Duttaphrynus ** | *atukoralei* | VUB 0101 | Sri Lanka | J882682 | FJ882629 | FJ882835-36 |
| *Duttaphrynus ** | *scaber* | SDB 532 | India: Western Ghats | J882683 | FJ882630 | FJ882785 |
| *Duttaphrynus ** | *parietalis* | SDB 10100 | India: Western Ghats | J882684 | FJ882631 | FJ882784 |
| *Duttaphrynus ** | sp. | SDB 927 | India: Western Ghats | J882685 | FJ882632 | FJ882792 |
| *Duttaphrynus* | *melanostictus* | VUB 0052 | India: Western Ghats | AY364167 | AY948805 | FJ882791 |
| *Duttaphrynus ** | *brevirostris* | SDB 4714 | India: Western Ghats | J882686 | FJ882633 | FJ882786 |
| *Duttaphrynus ** | sp. | SDB 4594 | India | J882687 | FJ882634 | FJ882839-40 |
| *Duttaphrynus ** | sp. | VUB 0680 | Vietnam | n.a. | n.a. | FJ882764-66 |
| *Duttaphrynus* | *himalayanus* | SDB 4566 | India | J882688 | FJ882635 | FJ882790 |
| *Duttaphrynus ** | *stuarti* | CAS 221485 | Myanmar | J882689 | FJ882636 | FJ882788 |
| *Duttaphrynus* | *crocus* | CAS 220193 | Myanmar | J882690 | FJ882637 | FJ882789 |
| “*Bufo” °* | *koynayensis* | SDB 2004-012 | India: Western Ghats | J882691 | FJ882638 | FJ882782 |
| *“Bufo” °* | sp. | SDB 4758 | India: Western Ghats | J882692 | FJ882639 | FJ882783 |
| *Adenomus* | *kelaartii* | VUB 0171 | Sri Lanka | EF107447 | EF107221 | FJ882780 |
| *Pedostibes* | *tuberculosus* | SDB 4691 | India: Western Ghats | J882693 | FJ882640 | FJ882793 |
| “Ansonia” ° | *ornata* | SDB 435 | India: Western Ghats | J882694 | FJ882641 | FJ882797 |
| *Ansonia* | *malayana* | KUHE 15472 | Malaysia ^gb^ | n.a. | n.a. | AB331712.1 |
| *Ansonia* | *hanitschi* | VUB 0615 | Malaysia: Borneo | J882695 | FJ882642 | FJ882794 |
| *Ansonia* | *spinulifer* | VUB 0647 | Malaysia: Borneo | J882696 | FJ882643 | FJ882798 |
| *Ansonia* | *fuliginea* | KUHE 17537 | Malaysia: Borneo ^gb^ | n.a. | n.a. | AB331709.1 |
| *Ansonia* | *leptopus* | VUB 0632 | Malaysia: Borneo | J882697 | FJ882644 | FJ882795 |
| *Ansonia* | *longidigita* | VUB 0666 | Malaysia: Borneo | J882698 | FJ882645 | FJ882796 |
| *Pelophryne* | sp. | KUHE 35585 | Malaysia ^gb^ | n.a. | n.a. | AB331720.1 |
| *Pelophryne* | *signata* | VUB 0583 | Malaysia: Borneo | J882699 | FJ882646 | FJ882801 |
| *Pelophryne* | *misera* | VUB 0641 | Malaysia: Borneo | J882700 | FJ882647 | FJ882800 |
| *Pelophryne* | *brevipes* | CMNHH 1617 | Philippines ^gb^ | n.a. | n.a. | AF375503, AF375530 |
| *Leptophryne* | *borbonica* | VUB 0673 | Malaysia: Borneo | EF107450 | EF107224 | FJ882799 |
| *Ingerophrynus* | *divergens* | VUB 0602 | Malaysia: Borneo | J882701 | FJ882648 | FJ882802 |
| *Ingerophrynus* | *galeatus* | ZMMSU A-3941 | Vietnam | J882702 | FJ882649 | FJ882767-68 |
| *Ingerophrynus* | *macrotis* | CAS 230357 | Myanmar | J882703 | n.a. | FJ882803 |
| *Bufo* | *bufo* | VUB 0982 | Belgium | J882704 | FJ882650 | FJ882806 |
| *Bufo* | *verrucosissimus* | ZMMSU A-4092 | Russia | J882705 | FJ882651 | FJ882807 |
| *Bufo* | *spinosus* | VUB 0517 | Spain | J882706 | FJ882652 | FJ882841-42 |
| *Bufo* | *gargarizans* | ZMMSU A-4127 | China | J882707 | FJ882653 | FJ882843-44 |
| *Bufo* | *andrewsi* | CAS 228184 | China | J882708 | FJ882654 | FJ882808 |
| *Epidalea* | *calamita* | VUB 0501 | France | J882709 | FJ882655 | FJ882809 |
| “Pedostibes” | *hosii* | VUB 0661 | Malaysia: Borneo | EF107449 | EF107223 | FJ882804 |
| *Phrynoidis* | *juxtaspera* | VUB 0649 | Malaysia: Borneo | J882710 | FJ882656 | FJ882805 |
| *Pseudepidalea* | cf. *surda* | ZMMSU A-4027 | Iran | J882711 | FJ882657 | FJ882810 |
| *Pseudepidalea* | cf. *pewzowi* | NP B-4-1 | Uzbekistan | J882712 | n.a. | FJ882811 |
| *Pseudepidalea* | cf. *variabilis* | VUB 1813 | Turkey | J882713 | FJ882658 | FJ882812 |
| *Pseudepidalea* | *viridis* | NP B-2-1 | Greece | J882714 | FJ882659 | FJ882813 |
| *Churamiti* | *maridadi* | MTSN 5584 | Tanzania | J882715 | FJ882660 | FJ882769-70 |
| *Nectophrynoides* | *minutus* | MW 3309 | Tanzania | n.a. | n.a. | FJ882814 |
| *Nectophrynoides* | *tornieri* | MW 1822 | Tanzania | EF107490 | EF107270 | FJ882815 |
| *Nectophrynoides* | *viviparus* | MW 1894 | Tanzania | J882716 | FJ882661 | FJ882816 |
| *Schismaderma* | *carens* | MW 4279 | Tanzania | J882717 | FJ882662 | FJ882849-50 |
| *“Pseudepidalea”* | *brongersmai* | VUB 1786 | Morocco | J882718 | FJ882663 | FJ882817 |
| *Wolterstorffina* | *parvipalmata* | MTSN 5895 | Cameroon | J882719 | FJ882664 | FJ882818 |
| *Werneria* | *mertensiana* | DPL 5107 | Cameroon ^gb^ | n.a. | n.a. | DQ283348 |

| Genus | Species | Voucher / Tissue Ref | Locality | Genbank | | |
| --- | --- | --- | --- | --- | --- | --- |
|  |  |  |  | CXCR4 | NCX1 | Mitochondrial DNA |
| INGROUP |  |  |  |  |  |  |
| *Mertensophryne* | *uzunguensis* | BM 2002.157 | Tanzania | J882720 | FJ882665 | FJ882819 |
| *Mertensophryne* | *taitana* | BM 2005.1540 | Tanzania | n.a. | n.a. | FJ882845-46 |
| *Mertensophryne* | *loveridgei* | KMH 26653 | Tanzania | J882721 | FJ882666 | FJ882820 |
| *Mertensophryne* | *lindneri* | BM 2002.394 | Tanzania | n.a. | FJ882667 | FJ882847-48 |
| *Mertensophryne* | *micranotis* | MW 3839 | Tanzania | EF107491 | EF107271 | FJ882821 |
| *Amietophrynus* | *brauni* | MW 3840 | Tanzania | EF107492 | EF107272 | FJ882822 |
| *Amietophrynus* | *poweri* | CAS 193857 | Namibia | J882722 | n.a. | FJ882771–73 |
| *Amietophrynus* | *garmani* | CAS 214829 | Kenya | J882723 | FJ882668 | FJ882823 |
| *Amietophrynus* | *gracilipes* | CAS 207620 | Equatorial Guinea | J882724 | FJ882669 | FJ882824 |
| *Amietophrynus* | *gutturalis* | MW 4174 | Tanzania | J882725 | FJ882670 | FJ882851-52 |
| *Amietophrynus* | *steindachneri* | CAS 214839 | Kenya | J882726 | FJ882671 | FJ882825 |
| *Amietophrynus ** | *mauritanicus* | NP B-22-1 | Morocco | J882727 | FJ882672 | FJ882826 |
| *Rhinella* | *granulosa* | VUB 1960 | Uruguay | J882728 | FJ882673 | FJ882774-75 |
| *Rhinella* | *schneideri* | VUB 1965 | Suriname | n.a. | FJ882674 | FJ882831 |
| *Rhinella* | *marina* | KU 289750 | El Salvador ^gb^ | DQ306535.1 | n.a. | DQ158497 |
| *Rhinella* | cf. *margaritifera* | MW 1006 | Guyana | J882729 | FJ882675 | FJ882832 |
| *Rhinella* | *nesiotes* | UTA 53310 | Bolivia ^gb^ | DQ306527.1 | n.a. | DQ158495 |
| *Rhinella* | *festae* | KU 217501 | Ecuador ^gb^ | DQ306500.1 | n.a. | DQ158478 |
| *Rhinella* | *veraguensis* | USNM 346048 | Peru ^gb^ | DQ306530.1 | n.a. | DQ158473 |
| *Rhinella* | *vellardi* | KU 211765 | Peru ^gb^ | DQ306541.1 | n.a. | DQ158433 |
| *Rhinella* | *atacamensis* | KU 217352 | Chile ^gb^ | DQ306521.1 | n.a. | DQ158423 |
| *Incilius* | *alvarius* | USNM 320001 | U.S.A ^gb^ | DQ306512.1 | n.a. | DQ158477 |
| *Incilius* | *luetkenii* | KU 289850 | El Salvador ^gb^ | DQ306501.1 | n.a. | DQ158461 |
| *Anaxyrus* | *americanus* | CAS 207258 | U.S.A | J882730 | FJ882676 | FJ882827 |
| *Anaxyrus* | *californicus* | CAS 175636 | U.S.A | n.a. | n.a. | FJ882828 |
| *Anaxyrus* | *terrestris* | CAS 207171 | U.S.A | J882731 | FJ882677 | FJ882829 |
| *Anaxyrus* | *boreas* | CAS 201586 | U.S.A | J882732 | FJ882678 | FJ882830 |
| *Rhaebo* | *nasicus* | ROM 20560 | Guyana ^gb^ | DQ306515.1 | n.a. | DQ158494 |
| *Rhaebo* | *haematiticus* | QCAZ 13215 | Ecuador ^gb^ | DQ306540.1 | n.a. | DQ158446 |
| *Nannophryne* | *variegata* | IZUA 3198 | Chile ^gb^ | DQ306496.1 | n.a. | DQ158420 |
| *Nannophryne* | *cophotis* | KU 211685 | Peru ^gb^ | DQ306565.1 | n.a. | DQ158467 |
| *Dendrophryniscus* | *minutus* | QCAZ 883 | Ecuador ^gb^ | DQ306516.1 | n.a. | DQ158425 |
| *Melanophryniscus* | *stelzneri* | VUB 0985 | South America ^‡^ | AY948784 | AY948822 | FJ882853 |
